# Could MDMA Promote Stemness Characteristics in Mouse Embryonic Stem Cells via mGlu5 Metabotropic Glutamate Receptors?

**Published:** 2012-12-12

**Authors:** Rokhsareh Meamar, Fereshte Karamali, Seyed Ali Mousavi, Hossein Baharvand, Mohammad Hossein Nasr-Esfahani

**Affiliations:** 1. Department of Cell and Molecular Biology, Cell Science Research Center, Royan Institute for Animal Biotechnology, ACECR, Isfahan, Iran; 2. Isfahan Neurosciences research center, Isfahan University of Medical Sciences, Isfahan, Iran; 3. Islamic Azad University, Najafabad Branch, Isfahan, Iran; 4. Department of Stem Cells and Developmental Biology, Cell Science Research Center, Royan Institute for Stem Cell Biology and Technology, ACECR, Tehran, Iran; 5. Department of Developmental Biology, University of Science and Culture, ACECR, Tehran, Iran

**Keywords:** Embryonic Stem Cells, Ecstasy, MDMA, Self Renewal, mGlu Receptor

## Abstract

**Objective:**

Ecstasy, or 3, 4 (±) methylenedioxymethamphetamine (MDMA), is a potent neurotoxic drug. One of the mechanisms for its toxicity is the secondary release of glutamate. Mouse embryonic stem cells (mESCs) express only one glutamate receptor, the metabotropic glutamate receptor 5 (mGlu5), which is involved in the maintenance and self-renewal of mESCs. This study aims to investigate whether MDMA could influence self-renewal via the mGlu5 receptor in mESCs.

**Materials and Methods:**

In this expremental study, we used immunocytochemistry and reverse transcription-polymerase chain reaction (RT-PCR) to determine the presence of the mGlu5 receptor in mESCs. The expression of mGlu5 was evaluated after MDMA was added to mESCs throughout neural precursor cell formation as group 1 and during neural precursor cell differentiation as group 2. The stemness characteristic in treated mESCs by immunofluorescence and flow cytometry was studied. Finally, caspase activity was evaluated by fluorescence staining in the treated group. One-way ANOVA or repeated measure of ANOVA according to the experimental design was used for statistical analyses.

**Results:**

In this study mGlu5 expression was shown in mESCs. In terms of neuronal differentiation, MDMA affected mGlu5 expression during neural precursor cell formation (group 1) and not during neural precursor differentiation (group 2). MDMA (450 µM) induced a significant increment in self-renewal properties in mESCs but did not reverse 2-methyl-6(phenylethynyl) pyridine (MPEP, 1 µM), a non-competitive selective mGlu5 antagonist. Fluorescence staining with anti-caspase 3 showed a significant increase in the number of apoptotic cells in the MDMA group.

**Conclusion:**

We observed a dual role for MDMA on mESCs: reduced proliferation and maintenance of self-renewal. The lack of decreasing stemness characteristic in presence of MPEP suggests that MDMA mediates its role through a different mechanism that requires further investigation. In conclusion, despite being toxic, MDMA maintains stemness characteristics.

## Introduction

Ecstasy or 3, 4 (±) methylenedioxymethamphetamine (MDMA) is a designer drug that has caused a worldwide dilemma due to its psychotic effects of euphoria, empathy, and increased self-esteem (1, 3). These drugs induce a rapid release of 5-hydroxytryptamine (5-HT) and dopamine (DA) from presynaptic vesicles and inhibit the reuptake of these neurotransmitters, which subsequently results in neuronal toxicity that may last for months in rats and years in primates and humans (4-7). Consequently DA motivates the release of glutamate, an excitatory amino acid that binds to the metabotropic glutamate receptor (mGlu5), which is coupled with the NMDA receptor to induce neurotoxicity by the release of calcium (8-11).

Ecstasy or MDMA is used primarily as a recreational drug during the child-bearing years. By using embryonic stem cells (ESCs) *in vitro* to examine pharmacologic and toxicological effects of drugs (12-15) we have shown that MDMA is a moderate or weak teratogen (16).

The only glutamate receptor expressed by ESCs is mGlu5, which is involved in the maintenance and self-renewal of mouse ESCs (mESCs). The differentiation of ESCs via formation of aggregates or embryoid bodies (EB) progressively decreases the expression of mGlu5 (17-19). According to previous studies MDMA, despite being a toxic drug, enhances the differentiation and survival of DA neurons both *in vivo* and *in vitro* (20, 21); however no report regarding the effects of this compound in the early embryonic stage has been published. Considering the role of MDMA in releasing glutamate and mGlu5 in the maintenance and self-renewal of mESCs, this study aims to investigate whether MDMA could influence self-renewal via the mGlu5 receptor.

## Materials and Methods

### Culture of ESCs

Royan B1(RB1) mESCs were cultured on inactivated embryonic fibroblasts in an ESC medium that contained Knockout™ Dulbecco’s Modified Eagle’s Medium (KDMEM, Gibco, 10829-018, Germany), supplemented with 15% ES-fetal calf serum (Gibco, 16141-079, Germany), 0.1 mM β-mercaptoethanol (Sigma-Aldrich, M7522, USA), 2 mM glutamine (Gibco, 15039-027, Germany), 0.1 mM non-essential amino acids (Sigma-Aldrich, M7145, USA), and 1000 IU/ml leukemia inhibitory factor (LIF, Chemicon, ESGRO, ESG1107, Germany) according to Baharvand et al. (22). In order to obtain neural differentiation, after EB formation with 1000 cells per 20 µl hanging drops for two days, we treated EBs with 1 µM retinoic acid (RA, Sigma-Aldrich, R2625, USA) for an additional four days in suspension. The EBs were individually plated on day six in 1% gelatin-coated wells of a 24-well tissue culture plate and allowed to grow for four days after plating, according to Meamar et al. (16).

### Experimental design

mESCs at a density of 2500 cells/cm^2^ were cultured on gelatin (0.1%, Sigma-Aldrich, G2500, USA) for four days in the presence of 450 µM MDMA (Sigma-Aldrich, M6403, USA), 1 µM 2-methyl-6 (phenylethynyl) pyridine (MPEP) a non-competitive selective mGlu5 antagonist (Sigma-Aldrich M5435, USA), MDMA/MPEP, and 30 µM glutamate (Sigma-Aldrich,G3291, USA). In another design, ESCs were treated with MDMA either throughout the process of neural differentiation (group 1), or only during post-plating (group 2). We assessed the ID50 for neuronal differentiation in groups 1 (60 µM) and 2 (130 µM) (16), and used the concentrations before and after ID50 for both groups.

### Immunocytochemistry and flow cytometry analysis

ESCs were fixed with 4% paraformaldehyde (Sigma-Aldrich, P6148, USA) and permeabilized with 0.2% Triton X100. Cells were then treated with 10 mg/ml bovine serum albumin (BSA; Sigma-Aldrich, A3311, USA) and incubated overnight at 4℃ with primary antibodies and anti-mouse IgG against mGlu5 (1:100, Abcam, ab53090) or anti-mouse IgM against SSEA1 (1:100, Chemicon, MAB4301, Germany). Cells were subsequently labeled with FITC-conjugated secondary antibody, goat anti-rabbit (1:80, Sigma-Aldrich, F1262, USA), and goat anti-mouse (1:125, Sigma-Aldrich, F9259, USA) for 60 minutes at room temperature, respectively. Nuclei were counterstained with DAPI (3 ng/ml, Sigma-Aldrich, D9564, USA) and cells were analyzed for mGlu5 expression with a fluorescent microscope (Olympus, BX51, Japan). Quantitative expression of SSEA1 was determined with a FACS Calibur flow cytometer (Becton Dickinson, San Jose, CA, USA) equipped with a 488 nm laser. For each sample, 10000 cells were calculated and single cells were selected by forward and side scatter gate from debris and aggregates. An isotype antibody was used as a negative control. Green fluorescence from the FITC-conjugated secondary antibody was collected in fluorescence detector 1 (FL-1) with a 530/30 nm band-pass filter. Each experiment was repeated in triplicate and the acquired data analyzed with CellQuest Pro software (23).

### Reverse transcription-polymerase chain reaction (RT-PCR) analysis

Total RNA was extracted using the RNeasy Mini Kit (Qiagen, Spain). RNA samples were then digested with DNase I (Fermentas; EN0521, USA) to remove any genomic DNA contamination. Reverse transcription (RT) was carried out using 2 µg total RNA, oligo (dT), and the RevertAidTM H Minus First Strand cDNA Synthesis Kit (Fermentas, K1622, USA) according to good manufacturing practice (GMP). Complementary DNA was exposed to PCR amplification using mouse-specific primers according to the following conditions: initial denaturation at 94℃ for 5 minutes, denaturation at 94℃ for 30 seconds, annealing at 55-70℃ for 45 seconds, extension for 45 seconds at 72℃ (35 cycles), and a final polymerization at 72℃ for 10 minutes. The PCRs were performed in triplicate. PCR products were electrophoresed in 1.7% agarose gels that contained ethidium bromide (10 µg/ml) and bands were visualized using a UV transilluminator (SynGene, Korea). Finally, the amount of cDNA normalized was based on β-tubulin mRNA expression using Gene Tools software to estimate the semi-quantitative expression of different mRNAs. Gels of three independent replicates were analyzed (24). The sequences of primers were: mGlu5 (forward: 5'-ATTGCAGCTGTGTTTGCC -3' and reverse: 5'-AGTGACAACCCCTAGGTTGG -3'); Oct3/4 (forward: 5'-CACGAGTGGAAAGCAACTCAG -3' and reverse: 5'-CTGGGAAAGGTGTCCCTGTAG -3'); and β-tubulin (forward: 5'-GTTCCCACGTCTCCACTTCTTC-3' and reverse: 5'-CCAGGTCATTCATGTTGCTCTC-3').

### Analysis of apoptosis induced by MDMA

MDMA-treated (450 µM) and untreated stem cells were assessed for caspase activity according to GMP (Chemicon, APT403, Germany) and visualized with a fluorescence microscope. The labeled fluoromethyl ketone peptide inhibitor of caspase-3 (FAM-DEVD-FMK) produces a green fluorescence when it covalently binds to active caspase (25).

### Statistical analyses

Studies utilized either a one-way ANOVA or repeated measure of ANOVA according to the experimental design. P<0.05 was considered statistically significant.

## Results

**Fig 1:**
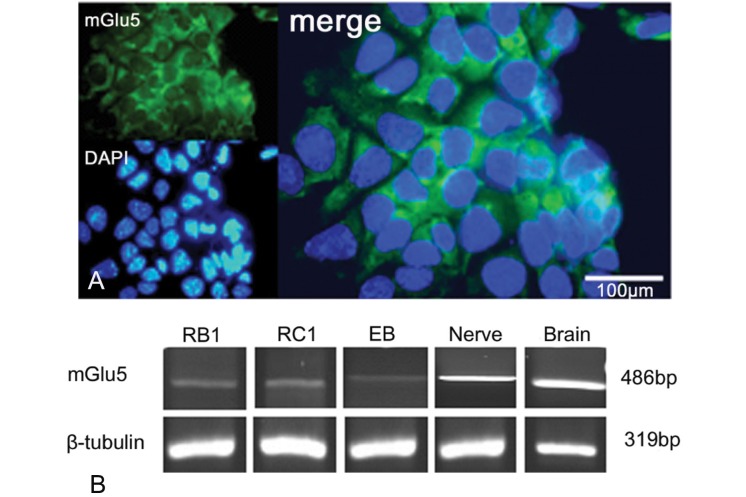
Expression of metabotropic glutamate receptor 5 (mGlu5) on embryonic stem cells (ESCs) using immunocytochemistry (A) and RT-PCR analysis (B). The nuclei were counterstained with DAPI.

**Fig 2:**
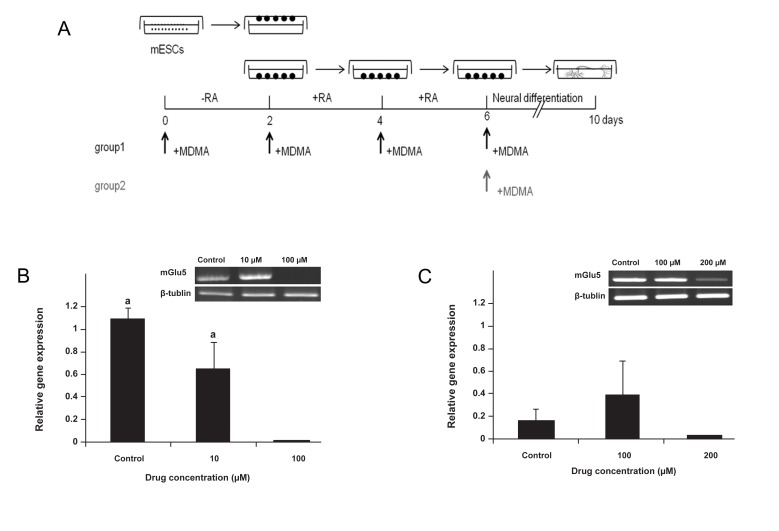
Experimental design for MDMA treatment (A). Embryonic stem cells (ESCs) were treated either with MDMA throughout the process of neural differentiation (group 1), or only during post-plating (group 2). B and C show semi-quantitative RT-PCR analyses of MDMA on the relative gene expression of metabotropic glutamate receptor 5 (mGlu5) in groups 1 (B) and 2 (C). a; P<0.002 for controls and 10 µM vs. 100 µM.

Figure 1A confirmed the presence of mGlu5 in the mESCs. As seen by RT-PCR analysis, the expression of mGlu5 increased when cells were treated with RA for neural differentiation and after plating when neurons were formed (Fig 1B). Treatment with 100 µM MDMA throughout the culture compared to the control and 10 µM MDMA significantly decreased the expression of mGlu5 (p<0.002). Treatment with 100 µM or 200 µM MDMA applied post-plating did not affect expression of mGlu5 (Fig 2).

**Fig 3:**
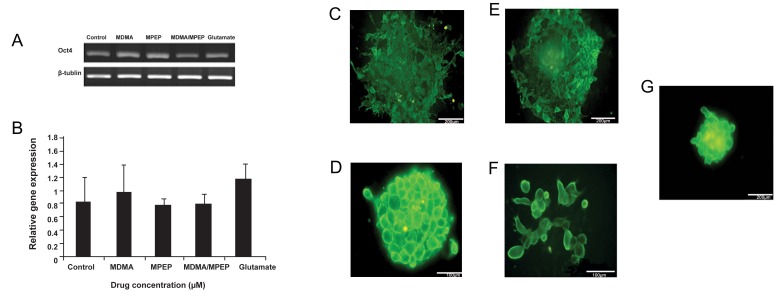
RT-PCR and semi-quantitative analyses of MDMA on the relative gene expression of Oct4 (A, B). Immunofluorescence staining of SSEA1 expression in control (C), and treated culture with MDMA (D), MPEP (E), MDMA/MPEP (F) and Glutamate (G).

**Fig 4:**
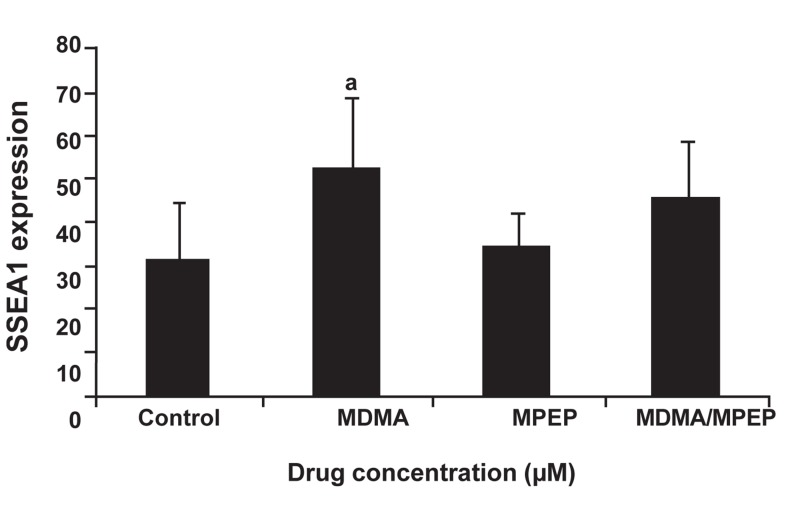
Percentage of cells expressing SSEA1 in control and after treatment with MDMA, MPEP, and MDMA/MPEP as assessed by flow cytometry. a; p<0.039 for MDMA vs. control.

**Fig 5:**
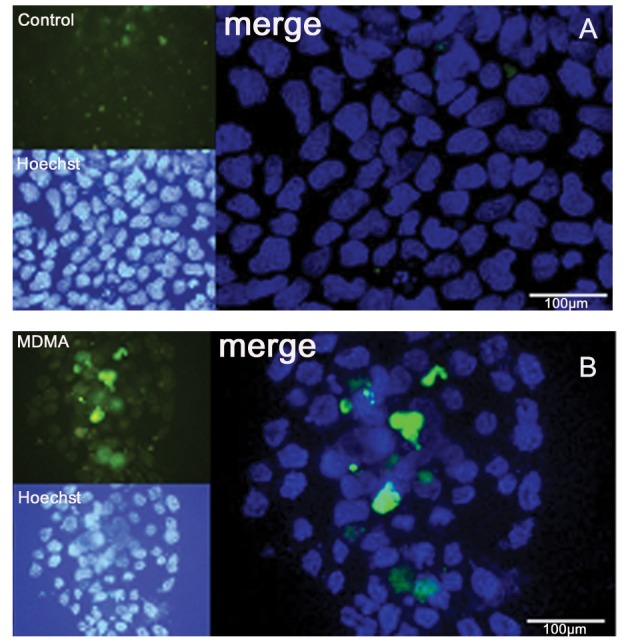
Fluorescence staining with anti-caspase 3 (FLICA) in control (A) and MDMA (B) groups for apoptosis. The nuclei were counterstained with Hoechst.

In order to evaluate the effect of MDMA on stemness characteristics, we treated stem cells with glutamate, MDMA at the IC50 of 450 µM, (16) or its antagonist MPEP in the presence of LIF and no feeder layer for four days. Cells were subsequently assessed for Oct4 expression, a marker of stem cells, by RT-PCR. The results showed no significant difference in RT-PCR level for the expression of Oct4 in the presence of glutamate, MDMA, and its antagonist MPEP (Figs 3A, B). To confirm these results, we assessed the expression of another stem cell marker, stage-specific embryonic antigen (SSEA1) by immunostaining. Despite the reduced number of colonies, the self-renewal of mESCs was maintained as shown by expression of SSEA1 (Fig 3D). Undifferentiated ESCs were presented with colonies, which were very prominent with a high nuclear-cytoplasmic ratio. SSEA1 expression declined when ESCs were treated with MPEP, a non-competitive selective mGlu5 receptor antagonist (Fig 3E). The addition of MPEP one hour before the addition of MDMA reduced the number of colonies, but not the expression of SSEA1 (Fig 3F). Adding glutamate (30 µM) also supported SSEA1 expression (Fig 3G). These observations were further confirmed by flow cytometry. The addition of MDMA prevented differentiation and significantly increased SSEA1 expression (MDMA vs. control, p<0.039); MPEP did not have any effect on this expression. The addition of MDMA to MPEP insignificantly reversed the effect of MDMA (Fig 4). Fluorescence staining with anti- caspase 3 showed a significant increase in the number of apoptotic cells in the MDMA group (60%) compared to the control group (15%; Fig5 A, B).

## Discussion

mGlu receptors are neurotransmitter receptors in the nervous system because they respond to synaptic glutamate and are involved in the regulation of synaptic plasticity (19, 26). However, the presence of this receptor in mESCs and its down-regulation during differentiation and up-regulation during neurogenesis suggests an additional role for mGlu receptors (27). For example, the endogenous activation of this receptor is necessary for maintenance of self-renewal properties in mESCs (17, 18). Initially the presence of mGlu5 has been investigated by immunostaining and RT-PCR analysis to prove its presence in mESCs. These observations are in agreement with previous reports that have confirmed the expression of mGlu5 on mESCs.

 Previous studies have suggested that expression of mGlu5 decreases during EB formation, followed by an increase during neurolation (17, 18). The results of mGlu5 in the current study are consistent with these reports. In this study we have also shown that treatment with the IC50 of MDMA (16) during neural precursor formation and differentiation significantly decreased expression of the mGlu5 receptor. However treatment with MDMA during neural differentiation (post-plating) did not affect the expression of mGlu5. These results have suggested that MDMA may affect mGlu5 expression during neural precursor cell formation but not during differentiation. It is important to note that in our previous study we have also shown that the IC50 of MDMA significantly decreased neural markers, MAP2 and nestin expression only during neural precursor cell formation (group 1) and not during differentiation (group 2) (16). Therefore, this suggests that MDMA may induce inhibition of neural precursor formation via down-regulation of the mGlu5 receptor, but this requires additional confirmation.

To further investigate the influence of this receptor and MDMA on stemness characteristics we applied glutamate, it’s natural agonist, during stem cell culture in the presence of LIF and absence of a feeder layer. The results revealed a higher expression of SSEA1. In a preliminary study we showed that glutamate depletion for 24 hours by glutamate transaminase reduced Oct4-GFP expression in mESCs. We showed that the IC50 dose of MDMA decreased the numbers of colonies despite the increased expression of SSEA1. These observations were further confirmed by flow cytometry. No substantial changes in Oct4 expression were noted, possibly because reduction of Oct4 requires more than four days (28). Therefore, these results may imply a binary role for MDMA: reducing ESCs proliferation while maintaining or supporting self-renewal characteristics. In concordance with our results, such a role has been described for DA neurons. Previous studies have shown that MDMA, despite being a toxic drug, enhances the differentiation and survival of DA neurons both *in vivo* and *in vitro* (20, 21). In other studies, such results have been obtained for glutamate in MSCs and neural progenitor cells in the brain (29, 30).

In order to confirm whether MDMA’s effects on stemness characteristic were mediated by the mGlu5 receptor, MPEP was applied prior to MDMA. MPEP partially reduced SSEA1 expression, which thereby suggested that this effect of MDMA was possibly mediated by the mGlu5 receptor in addition to other possible pathways. It has been reported that methamphetamines and ecstasy could motivate Janus kinas/signal transducers and activators of transcription (JAK/STAT) pathways; this effect is independent of the mGlu5 receptor and could not be reversed by MPEP (31-33).

Previous research has reported that MPEP, itself, inhibited stemness characteristics (17). In our experiment the expression of stemness markers in the MPEP group was similar to the culture of mESCs in the absence of a feeder layer and presence of LIF. The difference observed between the two studies could be attributed to different types of stem cells as the current study’s cells were feeder-dependent.

The reduced cell proliferation, despite the maintenance of stemness characteristics may be related to the toxic effect of MDMA. This effect might be induced by the activation of the caspase pathway, as observed by increased caspase activity in the MDMA group. It has been reported that MDMA can modulate an apoptotic pathway and induce oxidative stress both *in vivo* and *in vitro* (34, 35). A study of the literature suggests that the activation of the mGlu5 receptor via protein kinase c (PKC) inhibits glycogen synthase kinase-3β (GSK3-β) (36), thus mimicking the actions of the Wnt signaling pathway (37) which regulates SSEA1 expression (38, 39) by inhibiting β-catenin phosphorylation. It may be expected that the addition of glutamate and MDMA increases the level of β-catenin and decreases its phosphorylated form, thereby maintaining self-renewal. However, this proposed mechanism needs further investigation. This is the first report which shows that MDMA can increase stemness characteristic in mESCs, elucidating the pathway that begins with MDMA-induced self-renewal and may allow us to better understand how MDMA reduces normal neuron loss that occurs during development.

## Conclusion

In this study we reported the binary role for MDMA on mESCs; reduced proliferation and maintenance of self-renewal. Pharmacological blockade of the mGlu5 receptor with MPEP before addition of MDMA only partially reduced this effect. This suggests that MDMA mediated its role through a different mechanism, which requires further investigation. In conclusion, despite its toxicity, MDMA maintains stemness characteristics.
